# Chemoprevention of 4NQO-Induced Mouse Tongue Carcinogenesis by AKT Inhibitor through the MMP-9/RhoC Signaling Pathway and Autophagy

**DOI:** 10.1155/2022/3770715

**Published:** 2022-10-05

**Authors:** Panpan Yin, Jiahui Chen, Yanlin Wu, Feng Gao, Jinlin Wen, Wenbin Zhang, Ying Su, Xinyan Zhang

**Affiliations:** ^1^Beijing Institute of Dental Research, Beijing Stomatological Hospital & School of Stomatology, Capital Medical University, 4 Tiantanxili, Dongcheng District, Beijing 100050, China; ^2^Department of Periodontology, Tianjin Stomatological Hospital, 75 Dagu Road, Heping District, Tianjin 300041, China

## Abstract

Oral cancer (OC), the most common cancer in the head and neck, which has a poor prognosis, histopathologically follows a stepwise pattern of hyperplasia, dysplasia, and cancer. Blocking the progression of OC in the precancer stage could greatly improve the survival and cure rates. AKT protein plays a critical role in the signal transduction of cancer cells, and we found that AKT was overexpressed in human OC samples through analysis of TCGA database. Therefore, this study is aimed at investigating the chemopreventive effect of an AKT inhibitor (MK2206 2HCl) on OC. *In vivo*, we established a 4-nitroquinoline-1-oxide- (4NQO-) induced mouse tongue carcinogenesis model to investigate the potential chemopreventive effect of MK2206 2HCl on mouse OC resulting from 4NQO. The results showed that MK2206 2HCl could significantly reduce the incidence rate and growth of OC, inhibit the transformation of dysplasia to cancer in the 4NQO-induced mouse tongue carcinogenesis model, and simultaneously markedly suppress cell proliferation, angiogenesis, and mast cell (MC) infiltration in 4NQO-induced mouse tongue cancers. *In vitro*, our results revealed that MK2206 2HCl could also inhibit oral squamous cell carcinoma (OSCC) cell malignant biological behaviors, including cell proliferation, colony formation, cell invasion, and migration, while promoting apoptosis. Mechanistic studies revealed that MK2206 2HCl suppressed matrix metalloproteinase 9 (MMP-9) and RhoC expression and promoted autophagy gene LC3 II expression. In summary, our findings demonstrated the chemopreventive effect of MK2206 2HCl on the 4NQO-induced mouse tongue carcinogenesis model, which likely has an underlying mechanism mediated by the MMP-9/RhoC signaling pathway and autophagy.

## 1. Introduction

Oral cancer (OC), the sixth most common malignancy worldwide with an incidence of over 377,713 newly diagnosed cases and 177,757 deaths in 2020 [1, 2], is one of the major public health issues. Oral squamous cell carcinoma (OSCC) accounts for more than 90% of OC and usually develops from precancerous lesions such as oral leukoplakia and erythroplakia [3]. Its histopathology follows the gradual pattern of hyperplasia, dysplasia, and cancer [4]. Clinically, despite advances in surgery, radiotherapy, and chemotherapy, the five-year survival of OSCC patients is only approximately 50% [5, 6]. Remarkably, chemoprevention is a method to prevent, slow down, or reverse the process of cancer development in the early stage, thereby reducing the incidence and mortality of cancer. Therefore, it is very important to formulate chemoprevention strategies for OC.

AKT (protein kinase B), a key factor in the AKT/PI3K signaling axis, acts on regulating the survival, growth, and metastasis of cancer cells [7]. Studies have shown that excessive activation of AKT is a common molecular feature of human malignant tumors [8] and is found in approximately 40% of breast, ovarian, OC, and pancreatic cancers and more than 50% of prostate cancers [9, 10]. In addition, the overexpression of AKT was also related to cell drug resistance [11, 12], the degree of differentiation of cancer cells [13], and the patient survival rate [11, 14, 15]. Therefore, our study is aimed at exploring the chemopreventive effect of AKT inhibitors on OSCC.

MK2206 2HCl is an oral allosteric AKT inhibitor [16]. In a preclinical model, MK2206 2HCl enhanced the activities of molecular targeted drugs (lapatinib and erlotinib) and standard chemotherapy drugs (docetaxel, carboplatin, doxorubicin, and gemcitabine) in breast cancer, lung cancer, ovarian cancer, or hepatocellular carcinoma cell lines [16, 17]. Although MK2206 2HCl has antitumor activity for patients with advanced solid tumors, its side effects are mainly fatigue, grade 3 rash, grade 1-2 nausea and vomiting, and neutropenia [18–20], which limits the application of MK2206 2HCl in cancer treatment. However, there are no reports about the chemopreventive effect of MK2206 2HCl on the precancerous stage of cancer.

4-Nitroquinoline 1-oxide (4NQO), a synthetic water-soluble carcinogen, can induce tumors mainly in the tongue [21]. In this study, the 4NQO-induced mouse tongue carcinogenesis model was established to investigate the chemopreventive effect of MK2206 2HCl on OC. We also explored the inhibitory effect of MK2206 2HCl on OSCC cell lines *in vitro*. Further mechanistic studies were also conducted to delve into the underlying mechanism.

## 2. Materials and Methods

### 2.1. Cell Culture

The CAL27 OSCC cell line was provided as a gift by Wuhan University. SCC25 cells were purchased from the American Type Culture Collection (ATCC, Manassas, VA). All cells were cultured in Dulbecco's modified Eagle's medium (DMEM/high glucose, Gibco, Grand Island, NY) supplemented with 10% fetal bovine serum (FBS, Gibco), 100 *μ*g/mL streptomycin, and 100 U/mL penicillin and incubated at 37°C with 5% CO_2_.

### 2.2. Real-Time PCR

Total RNA was extracted from cells by using TRIzol (ComWin Biotech Co., Ltd, Beijing, China). cDNA was synthesized by a reverse transcription kit (ComWin). RT-PCR analysis of gene expression was performed with 1 *μ*L of cDNA and SYBR Mixture (ComWin). The RT-PCR results were expressed by the 2^-*ΔΔ*Ct^ method. Specific primer sets were as follows: MMP-9 (forward, 5′-3′TGTACCGCTATGGTTACACTCG; reverse, 5′-3′GGCAGGGACAGTTGCTTCT), RhoC (forward, 5′-3′GGAGGTCTACGTCCCTACTGT; reverse, 5′-3′CGCAGTCGATCATAGTCTTCC), and GAPDH (forward, 5′-3′CATGGGTGTGAACCATGAGAAGTAT; reverse, 5′-3′GACTGTGGTCATGAGTCCTTCCA).

### 2.3. Western Blotting

Cells were lysed by RIPA buffer. Subsequently, the protein was quantified and denatured. Western blotting was performed according to the standard protocol. Protein samples (25 *μ*g/well) were separated by 10% SDS-PAGE (Bio-Rad, Hercules, CA) and electroblotted onto PVDF membranes (Bio-Rad), which were then blocked with 5% skimmed milk and incubated with primary antibodies (anti-AKT, 1 : 5000, ab32505, Abcam, Cambridge, MA; anti-phospho-AKT (S473), 1 : 5000, ab81283, Abcam; anti-MMP-9, 1 : 1000, ab137867, Abcam; anti-RhoC, 1 : 1000, ab180785, Abcam; anti-p-Rb, 1 : 1000, CST, 9307S; anti-p21, 1 : 1000, 10355-1-AP, Proteintech, Chicago, IN; anti-LC3, 1 : 1000, A7198, ABclonal, Wuhan, China; and anti-GAPDH, 1 : 2500, M121107, HuaAn Biotechnology, Hangzhou, China). Protein bands were visualized with a chemiluminescence (ECL) reagent (Bio-Rad).

### 2.4. Proliferation Assay

OSCC cells (6000/well) were seeded in 96-well plates, and MK2206 2HCl (2, 4, 6, 8, and 10 *μ*M) was added (Selleck Chemicals, Houston, TX). After treatment with MK2206 2HCl for 24 h, 48 h, and 72 h, MTT (Sigma-Aldrich, Saint Louis, MO) solution (5 mg/mL, 20 *μ*L) was added to the cells and cultivated at 37°C, 5% CO_2_ for 4 h. Finally, the purple crystals were dissolved by treatment with 200 *μ*L DMSO (Sigma). The absorbance of the reactants was recorded at 490 nm using the microplate reader (Molecular Devices, Sunnyvale, CA).

### 2.5. Colony Formation Assay

After treatment with MK2206 2HCl (6 *μ*M and 10 *μ*M) for 24 h, OSCC cells were seeded in 60 mm diameter plates (500/plate). After culturing for 12 days, methanol was used to fix the colonies for 10 min, and crystal violet staining solution (Beyotime, Xiamen, China) was used to stain the colonies for 30 min.

### 2.6. Cell Migration Assay

Cells were plated into 6-well plates. Then, a 200 *μ*L pipette tip was used to make a straight wound, and cell debris was removed by PBS. Finally, MK2206 2HCl (2 *μ*M, 6 *μ*M, and 10 *μ*M) was added to the cells. Photographs were obtained at 0, 24 h, and 48 h under a microscope (OLYMPUS, IX71, Tokyo, Japan). Measuring the scratch area was our method to evaluate the migration rate.

### 2.7. Transwell Invasion Assay

First, the cells were treated with MK2206 2HCl (2 *μ*M, 6 *μ*M, and 10 *μ*M) for 24 h. For the Transwell invasion assay, Matrigel (Corning, NY) was loaded on the Transwell membranes (8 *μ*m pore size; Corning). Subsequently, OSCC cells (4.5 × 10^5^/well) in serum-free DMEM were inoculated into the top chamber. Medium containing 10% FBS was added to the bottom chamber. After 48 h of culture, cotton balls were used to remove the cells that remained on the top membrane. Finally, methanol and crystal violet staining solution (Beyotime) were used to fix and stain the cells that had passed through the membrane. The number of passed cells was counted under a microscope (OLYMPUS, IX71).

### 2.8. Immunofluorescence Analysis

After treatment with MK2206 2HCl (6 *μ*M and 10 *μ*M) for 24 h, OSCC cells were fixed with methanol for 15 min and incubated with primary antibodies against LC3B (A7198, ABclonal, 1 : 100) and p62 (A7758, ABclonal, 1:200) overnight at 4°C. Subsequently, samples were incubated with the secondary antibody (A11035, Thermo Fisher, Waltham, MA, 1 : 500) for 30 min and then incubated with DAPI (Sigma) for 5 min. Finally, the positive signal was acquired by microscopy (OLYMPUS, BX61).

### 2.9. Cell Cycle Analysis

After treatment with various concentrations of MK2206 2HCl (6 *μ*M and 10 *μ*M) for 24 h, cells were harvested, fixed, and placed at 4°C overnight. Finally, the cell nucleus was stained with solution containing RNase (100 *μ*g/mL) (Solarbio, Beijing, China) and propidium iodide (10 *μ*g/mL) (Solarbio) for 30 minutes in the dark. A flow cytometer (BD, Accuri C6, Franklin Lake, NJ) was used to analyze a total of 10,000 stained nuclei. The cell cycle results were evaluated using Modfit LT software (Verity Software House, Topsham, ME).

### 2.10. Apoptosis Assays

Apoptosis was assessed by flow cytometric analysis using PE-Annexin V and 7-AAD staining. Briefly, cells were harvested by free-EDTA-trypsinization (Gibco) and resuspended in 1x budding buffer (BD). At room temperature, we incubated the cells with PE-Annexin V (BD) for 20 min. Then, the cells were incubated with 7-AAD (BD) for 20 min in the dark. Finally, the cells were evaluated by flow cytometry (BD, Accuri C6).

### 2.11. Establishment of 4NQO-Induced Mouse Tongue Carcinogenesis Model

Fifty-seven C57BL/6 mice (6-7 weeks; mean weight 20.5 g) were purchased from the SPF (Beijing) Biotechnology Co., Ltd. (Beijing, China). After being maintained in a specific-pathogen-free animal facility for one week, all mice were divided into three groups: group A mice (20/57: blank control group) received normal water; group B (18/57: 4NQO+captisol) and group C (19/57: 4NQO+MK2206 2HCl) mice received 4NQO (100 *μ*g/mL, Sigma) in their water daily for 10 weeks. From week 11, group B mice were gavaged with 15% captisol (AbMole, Houston, TX) and group C mice were gavaged with MK2206 2HCl (Selleck, 50 mg/kg, dissolved in 15% captisol) twice a week for 5 weeks. The body weights of all mice were measured once a week. All mice were euthanized at 24 weeks by the intraperitoneal injection of pentobarbital sodium (Merck, Darmstadt, Germany, 100 mg/kg). Tongue tissues were preserved in 4% paraformaldehyde for histopathological analysis.

### 2.12. Histopathology and Immunohistochemistry

Mouse tongue tissues were stained with H&E and analyzed by microscopy (OLYMPUS, BX61). For immunohistochemistry (IHC), sections were incubated with 0.01 mol/L citrate buffer (pH = 6) for 20 minutes in a microwave oven for antigen retrieval after deparaffinization. Sections were incubated with primary antibodies against phospho-AKT (S473) (ab81283, Abcam, 1 : 1000), p21 (10355-1-AP, Proteintech, 1 : 200), MMP-9 (ab137867, Abcam, 1 : 500), RhoC (ab180785, Abcam, 1 : 200), Ki67 (A00052208, DAKO, Denmark, 1 : 200), CD31 (ab76533, Abcam, 1 : 1000), and LC3 (A7198, ABclonal, 1 : 100). Samples were then incubated with secondary antibody (kit-5020, Maixin) for 20 minutes. Using Image-Pro Plus 6.0 software, we analyzed the results of IHC with a mean optical density (MOD = integral optical density value (IOD)/area of the whole) value [22].

### 2.13. Mast Cell (MC) Staining

After normal deparaffinization, toluidine blue staining solution (Solarbio) was used to stain the samples for 20 minutes. A high-power objective field (×400) was used to count the number of MC by microscopy (OLYMPUS, BX61).

### 2.14. Bioinformatics Analysis

We analyzed the expression of AKT in OC in TCGA database (https://www.cancer.gov/about-nci/organization/ccg/research/structural-genomics/tcga). Following searching the OC parameters, 341 OC and 30 normal tissue raw data were obtained. Then, the TPM value was standardized, and R v3.6.1 was used to draw the plots.

### 2.15. Statistical Analysis

Tumor incidence rates were analyzed by Fisher's exact test. Comparisons among multiple groups were evaluated through one-way ANOVA. *P* < 0.05 was considered the threshold of significance.

## 3. Results

### 3.1. AKT Expression in OC Tissues Evaluated in TCGA Datasets

**Figure 1:**
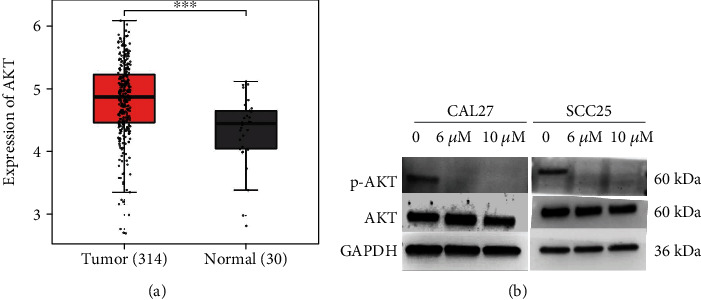
Assessment of AKT expression in oral cancer patients analyzed in TCGA datasets. (a) The expression levels of AKT in oral tumor tissues were higher than those in normal tissues analyzed in TCGA datasets. ^∗∗∗^*P* < 0.001 as compared with the normal tissues. (b) Protein expression of AKT and p-AKT in CAL27 and SCC25 cells after MK2206 2HCl treatment for 24 h by western blotting.

We analyzed the expression of AKT in OC in TCGA database. As evaluated in datasets from TCGA, AKT expression was significantly higher in OC tissues (*n* = 314) than in normal tissues (*n* = 30) (Figure 1(a)). This result demonstrated that AKT was overexpressed in OC tissues.

MK2206 2HCl, an AKT specific inhibitor, was subsequently used in animal and *in vitro* studies. First, we detected the inhibitory effect of MK2206 2HCl on AKT in OSCC cells. As expected, the western blotting results showed that p-AKT expression was decreased, but AKT expression remained almost unaltered in CAL27 and SCC25 cells after treatment with MK2206 2HCl (6 *μ*M and 10 *μ*M) for 24 h (Figure 1(b)).

### 3.2. Chemopreventive Effect of MK2206 2HCl on 4NQO-Induced Mouse Tongue Carcinogenesis Model

**Figure 2:**
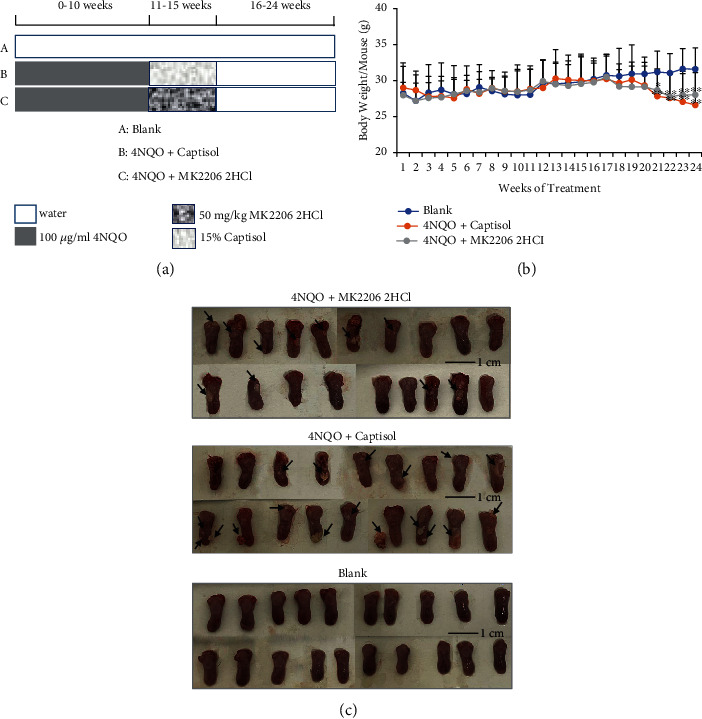
The applications and effects of MK2206 2HCl on the 4NQO-induced mouse oral carcinogenesis model. (a) Experimental protocol for the chemopreventive effect of MK2206 2HCl on 4NQO-induced mouse oral carcinogenesis model. (b) Mouse body weight. ^∗∗^*P* < 0.01,  ^∗^*P* < 0.05 as compared with the blank group. (c) Gross tumors and lesions in mouse tongues at the end of the 24th week. The tumors are indicated by arrows. Bar = 1 cm.

Over the course of this study (Figure 2(a)), the body weights of all mice were similar, without significant differences before week 20. Nevertheless, compared with the blank control group, the body weights of 4NQO-drinking mice were decreased significantly starting from the 21st week, which may be related to the development and progression of oral tumors (Figure 2(b)). The gross tumors and lesions in mouse tongues at the end of the 24th week are shown in Figure 2(c). The blank group mice had normal tongue mucosa with a pink, soft, smooth, and elastic tongue, without white spots and mass formation. However, the tongue mucosa of 4NQO-drinking mice was slightly white and rough, and some of them showed 1-2 mm white spots and/or exogenous papillary lumps. Compared with the 4NQO+captisol group (94.4%), MK2206 2HCl significantly reduced the visible tumor incidence rate (57.9%) in the 4NQO+MK2206 2HCl group (Table 1). The mean tumor volume also decreased from 4.05 mm^3^ in the 4NQO+captisol group to 3.01 mm^3^ in the 4NQO+MK2206 2HCl group, upon gross examination (Table 1).

**Figure 3:**
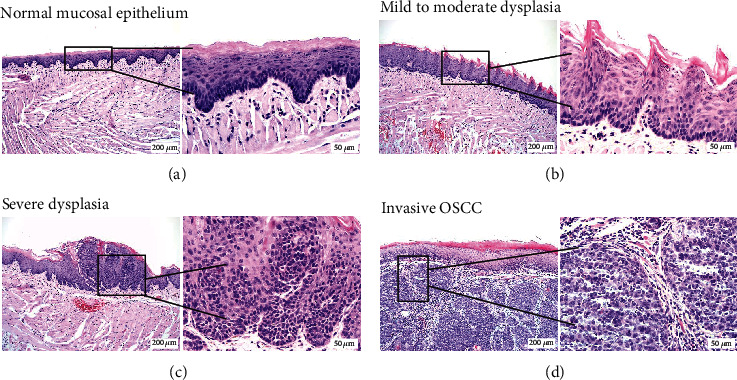
Histological examination of mouse tongue mucosa: (a) normal mouse tongue mucosal epithelium; (b) mouse tongue mucosal epithelium with mild to moderate dysplasia; (c) mouse tongue mucosal epithelium with severe dysplasia; (d) mouse tongue mucosal epithelium with invasive squamous cell carcinoma. Magnification ×100; ×400.

Representative images of the tongue mucosal epithelium from the blank, 4NQO+captisol, and 4NQO+MK2206 2HCl groups are shown in Figures 3(a)–3(d) by H&E staining.  Figure 3(a) was the normal tongue mucosal epithelium. The mice that were administered 4NQO in drinking water for 10 weeks exhibited hyperkeratosis, mild to moderate dysplasia (Figure 3(b)), severe dysplasia (Figure 3(c)), and invasive OSCC (Figure 3(d)). Briefly, total dysplasia rates were increased from 27.8% in the 4NQO+captisol group to 63.2% in the 4NQO+MK2206 2HCl group (Table 1). However, the squamous cell carcinoma incidence rates of 4NQO-induced mouse OC decreased from 72.2% in the 4NQO+captisol group to 36.8% in the 4NQO+MK2206 2HCl group, as shown by H&E staining (Table 1).

These results indicated the chemopreventive effect of MK2206 2HCl through inhibiting the incidence rates and growth of OC and simultaneously greatly suppressed the transformation of dysplasia to cancer in the 4NQO-induced mouse tongue carcinogenesis model.

### 3.3. The Effect of MK2206 2HCl on Cancer Cell Proliferation in 4NQO-Induced Mouse Tongue Carcinogenesis Model

**Figure 4:**
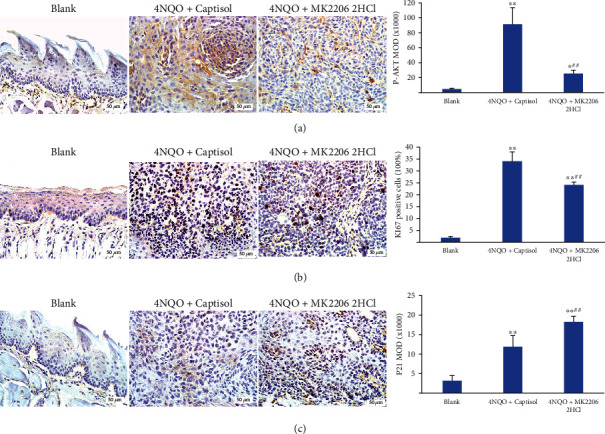
MK2206 2HCl suppressed cancer cell proliferation in 4NQO-induced mouse tongue tumors. (a) MK2206 2HCl inhibited p-AKT expression in 4NQO-induced mouse tongue tumors. (b) Cell proliferation was suppressed by MK2206 2HCl as showed by Ki67 positive cells. (c) MK2206 2HCl promoted p21 expression in tongue tumors. All data are presented as the mean ± SD. ^∗∗^*P* < 0.01,  ^∗^*P* < 0.05 as compared with the blank group, ^##^*P* < 0.01 as compared with the 4NQO+captisol group. Magnification ×400.

First, p-AKT expression in 4NQO-induced mouse tongue tumors was examined through IHC. The results showed that p-AKT expression in the 4NQO+MK2206 2HCl group was markedly reduced compared with that in the 4NQO+captisol group (Figure 4(a)). Afterward, the mechanism of MK2206 2HCl on 4NQO-induced epithelial proliferation was investigated. Ki67 nuclear staining is a marker of cell proliferation, and it was found that Ki67 expression in the 4NQO+MK2206 2HCl group was remarkably reduced compared to that of the 4NQO+captisol group through IHC staining (Figure 4(b)). In addition, p21, one of the cyclin-dependent kinase inhibitor families, is closely related to the cell cycle. Our IHC staining results revealed that p21 expression in the 4NQO+MK2206 2HCl group was markedly increased compared with that in the 4NQO+captisol group (Figure 4(c)).

These results confirmed the chemopreventive effect of MK2206 2HCl on OC cell proliferation in the 4NQO-induced mouse tongue carcinogenesis model.

### 3.4. The Effect of MK2206 2HCl on Angiogenesis and MC Infiltration of Cancers in 4NQO-Induced Mouse Tongue Carcinogenesis Model

**Figure 5:**
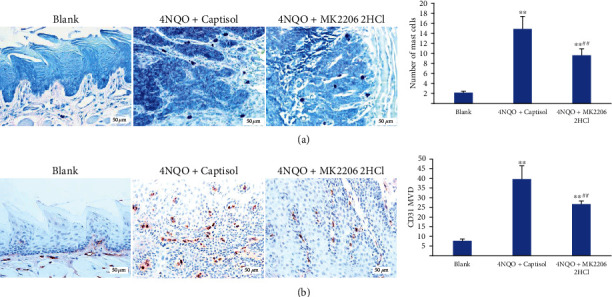
MK2206 2HCl suppressed MC infiltration and microvascular density in 4NQO-induced mouse tongue tumors. (a) MK2206 2HCl inhibited MC infiltration by toluidine blue staining. (b) MK2206 2HCl inhibited the microvascular density by IHC. All data are presented as the mean ± SD. ^∗∗^*P* < 0.01 as compared with the blank group, ^##^*P* < 0.01 as compared with the 4NQO+captisol group. Magnification ×400.

Mast cells, the key host cells for tumor invasion, are closely related to tumor growth and angiogenesis by releasing angiogenic factors. Therefore, we detected MC infiltration and microvascular density in 4NQO-induced mouse tongue cancers. Our results showed that MK2206 2HCl could decrease the MC infiltration number from 29.8 per field (×400) of the 4NQO+captisol group to 19.3 per field (×400) in the 4NQO+MK2206 2HCl group by toluidine blue staining (Figure 5(a)). Moreover, the microvascular density was also significantly reduced to 26.7 per field (×400) in the 4NQO+MK2206 2HCl group from 39.7 per field (×400) in the 4NQO+captisol group (Figure 5(b)).

These results confirmed the chemopreventive effect of MK2206 2HCl on angiogenesis and MC infiltration of cancers in the 4NQO-induced mouse tongue carcinogenesis model.

### 3.5. MK2206 2HCl Suppressed OSCC Cell Proliferation and Colony Formation

**Figure 6:**
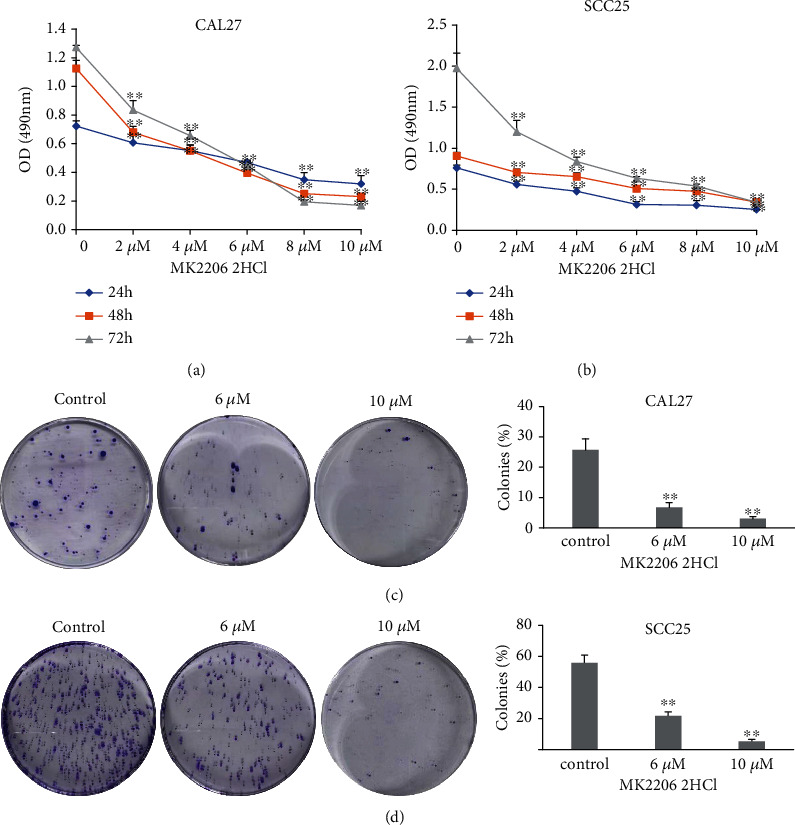
Growth-inhibitory effect of MK2206 2HCl on OSCC cell lines. (a) MK2206 2HCl treatment inhibited CAL27 cell proliferation, determined by MTT assay. (b) MK2206 2HCl treatment inhibited SCC25 cell proliferation, determined by MTT assay. (c) Colony formation assay showed that MK2206 2HCl inhibited colony formation of CAL27 cells. (d) The colony formation assay showed that MK2206 2HCl inhibited colony formation of SCC25 cells. All data are presented as the mean ± SD. ^∗∗^*P* < 0.01 as compared with the control group.

To detect the biological function of MK2206 2HCl in OSCC cells *in vitro*, we performed MTT and colony formation assays. OSCC cell lines CAL27 and SCC25 were treated with MK2206 2HCl (0, 2, 4, 6, 8, and 10 *μ*M) for 24, 48, and 72 hours. As shown in Figures 6(a) and 6(b), exposure to MK2206 2HCl led to dose- and time-dependent inhibition of cell viability. Consistently, the colony formation assay showed that MK2206 2HCl treatment dose-dependently markedly decreased the number of colonies in both CAL27 and SCC25 cells (Figures 6(c) and 6(d)). These data indicated that AKT inhibition with MK2206 2HCl could greatly suppress OSCC cell proliferation and colony formation abilities *in vitro*.

### 3.6. MK2206 2HCl Induced OSCC Cell Cycle Arrest at G2/M Phase

**Figure 7:**
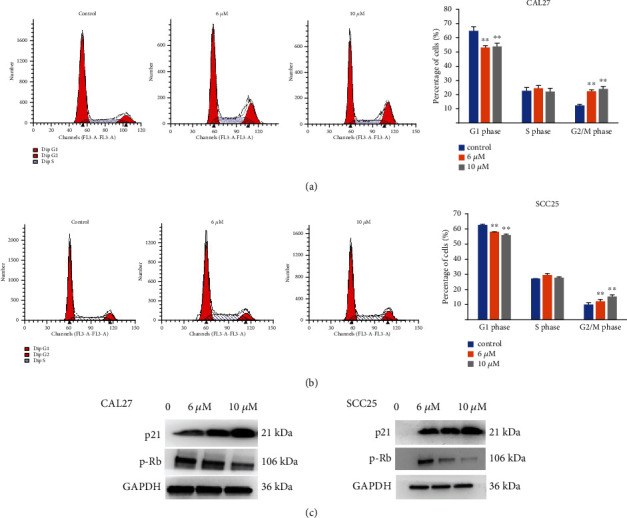
MK2206 2HCl induces cell cycle arrest at G2/M in OSCC cell lines. (a) MK2206 2HCl induces cell cycle arrest at G2/M in CAL27 cells. (b) MK2206 2HCl induces cell cycle arrest at G2/M in SCC25 cells. (c) MK2206 2HCl increased the protein expression of p21 and inhibited p-Rb expression by western blotting in CAL27 and SCC25 cells. All data are presented as the mean ± SD. ^∗∗^*P* < 0.01 as compared with the control group.

The effects of MK2206 2HCl on cell cycle progression were determined by flow cytometry in the OSCC cell lines CAL27 and SCC25. After treatment with MK2206 2HCl at various concentrations for 24 hours, the results revealed that the percentage of cells was dose-dependently increased in the G2/M phase and that the number of cells in the G1 phase was decreased accordingly by flow cytometry as shown in Figures 7(a) and 7(b). p-Rb is an oncogene that regulates the cell cycle by regulating the control points in the G1 phase. Compared with the control group, MK2206 2HCl also inhibited p-Rb expression and increased p21 expression in CAL27 and SCC25 cells, as shown by western blotting, which was consistent with the cell cycle results (Figure 7(c)).

These data indicated that AKT inhibition with MK2206 2HCl could induce cell cycle arrest via decreased p-Rb expression and increased p21 expression.

### 3.7. MK2206 2HCl Promoted OSCC Cell Apoptosis

**Figure 8:**
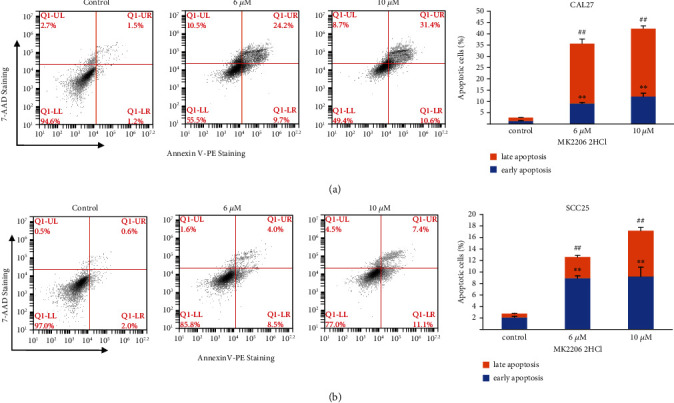
MK2206 2HCl promoted the apoptosis of OSCC cells. (a) MK2206 2HCl treatment for 24 h promoted the early and late apoptosis of CAL27 cells. (b) MK2206 2HCl treatment for 24 h promoted the early and late apoptosis of SCC25 cells. All data are presented as the mean ± SD. ^∗∗^*P* < 0.01 indicated early apoptotic cells compared with the control group. ^##^*P* < 0.01 as the late apoptotic cells compared with the control group.

To detect the effect of MK2206 2HCl on cell apoptosis, Annexin V and 7-AAD staining was performed in OSCC cells. After treatment with MK2206 2HCl for 24 hours, the results showed that the early and late apoptosis rates of CAL27 cells were dose-dependently increased by flow cytometry as shown in Figure 8(a). Similarly, apoptosis assays were also detected in SCC25 cells, and the results were consistent with CAL27 cells (Figure 8(b)). These results further demonstrated that MK2206 2HCl plays a tumor suppressor function by promoting apoptosis.

### 3.8. MK2206 2HCl Suppressed OSCC Cell Migration and Invasion

**Figure 9:**
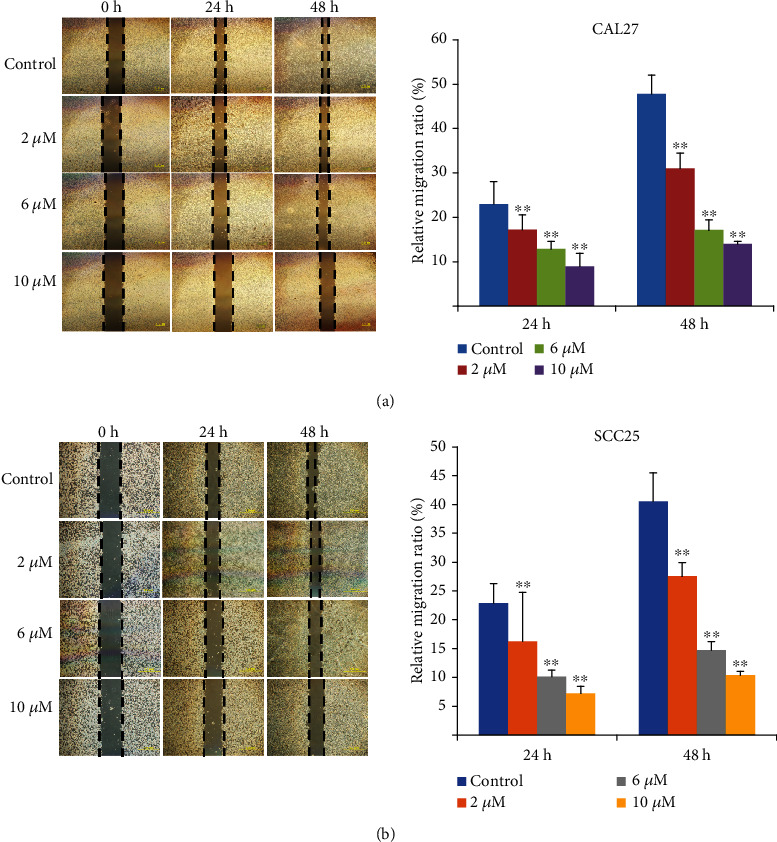
MK2206 2HCl suppressed the OSCC cell migration ability. (a) MK2206 2HCl inhibited the CAL27 cell migration ability by scratch migration assay. (b) MK2206 2HCl inhibited SCC25 cell migration ability by scratch migration assay. All data are presented as the mean ± SD. ^∗∗^*P* < 0.01 as compared with the control group. Magnification ×200.

**Figure 10:**
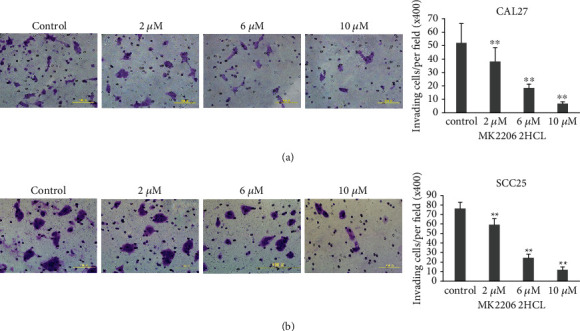
MK2206 2HCl suppressed the OSCC cell invasion ability. (a) MK2206 2HCl inhibited CAL27 cell invasive ability by Transwell invasion assay. (b) MK2206 2HCl inhibited SCC25 cell invasive ability by Transwell invasion assay. All data are presented as the mean ± SD. ^∗∗^*P* < 0.01 as compared with the control group. Magnification ×400.

To verify the effect of MK2206 2HCl on OSCC cell migrative and invasive abilities, migration and invasion assays were performed. Our results showed that compared to the control group, MK2206 2HCl inhibited the migration of the OSCC cell line CAL27 in a dose-dependent manner (Figure 9(a)). In addition, invasion assays evaluated that MK2206 2HCl markedly reduced the number of invading cells from 52.1 per field (×400) in the control group to 38.3 per field (×400) in the 2 *μ*M group, 18.4 per field (×400) in the 6 *μ*M group, and 6.8 in the 10 *μ*M group at 48 h (Figure 10(a)). As shown in Figures 9(b) and 10(b), migration and invasion assays were also detected in the OSCC cell line SCC25, which was consistent with CAL27 cells. These data further indicated that the AKT inhibitor MK2206 2HCl could inhibit the migration and invasion abilities of OSCC cells.

### 3.9. MK2206 2HCl Promoted the Autophagy in OSCC

**Figure 11:**
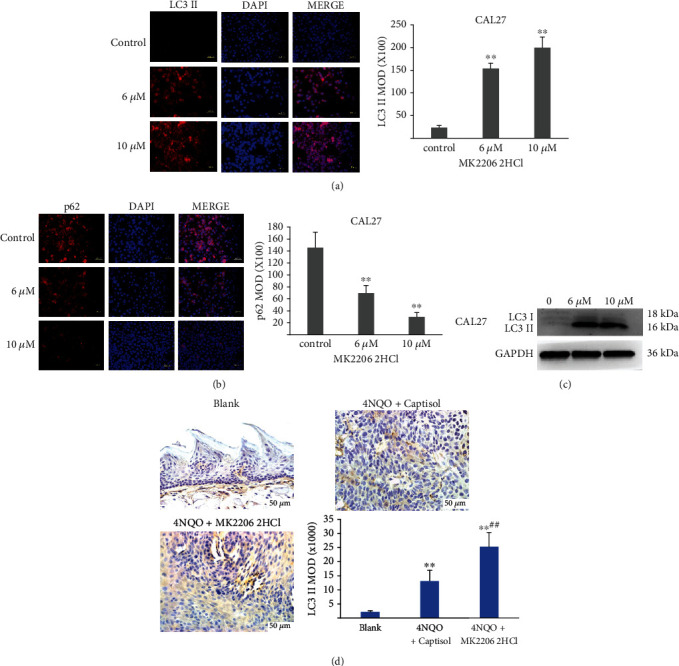
MK2206 2HCl induced LC3 II and p62 expressions in OSCC cells and 4NQO-induced mouse tongue tumors. (a) MK2206 2HCl promoted LC3 II expression in CAL27 cells as showed by immunofluorescence. Magnification ×400. (b) MK2206 2HCl decreased p62 expression in CAL27 cells. All data are presented as the mean ± SD. ^∗∗^*P* < 0.01 as compared with the control group. Magnification ×400. (c) The level of LC3 II was increased by western blotting in MK2206 2HCl-treated CAL27 cells. (d) MK2206 2HCl promoted LC3 II expression in 4NQO-induced mouse tongue tumors by IHC. All data are presented as the mean ± SD. ^∗∗^*P* < 0.01 as compared with the blank group, ^##^*P* < 0.01 as compared with the 4NQO+captisol group. Magnification ×400.

Since the PI3K/AKT signaling axis is related to autophagy activity, it may be another possible mechanism by which MK2206 2HCl suppresses tumor growth. Therefore, we explored the effect of MK2206 2HCl on autophagy in OSCC cells and 4NQO-induced mouse tongue tumors. Microtubule-associated protein 1 LC3 is an essential protein of autophagy. LC3 is processed from the full-length protein (MAP-LC3 I) into a cleaved and lipidated form (LC3 II), and LC3 II is considered to be an autophagy-specific marker. The conversion of LC3 I to LC3 II showing an upward trend was revealed by our western blotting results in OSCC cell treatment with MK2206 2HCl for 24 h (Figure 11(c)). In addition, LC3 II expression was significantly increased in CAL27 cells, as shown by the MOD value determined by immunofluorescence (Figure 11(a)). We further tested LC3 II expression *in vivo*, and the results showed that compared with the 4NQO+captisol group, MK2206 2HCl could also promote LC3 II expression in the 4NQO+MK2206 2HCl group through IHC staining (Figure 11(d)). LC3 II could bind to the adaptor protein p62 sequestosome, which is selectively degraded by autophagy. And p62 expression was significantly decreased in CAL27 cells by immunofluorescence (Figure 11(b)). Similar results were also obtained in the SCC25 cells, where MK2206 2HCl promoted autophagy in SCC25 cells (Supplemental Figure 1).

These data demonstrated that the chemopreventive effect of MK2206 2HCl on OSCC may induce autophagy by regulating LC3 II and p62 expressions.

### 3.10. MK2206 2HCl Suppressed MMP-9 and RhoC Expression in OSCC

**Figure 12:**
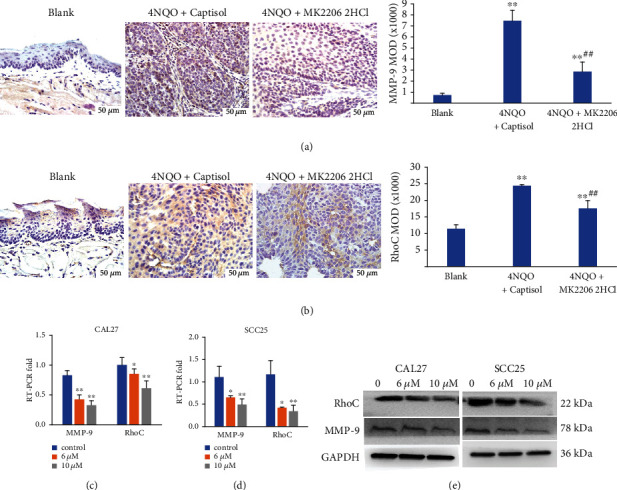
Inhibitory effects of AKT inhibitor on MMP-9/RhoC expression. (a) MK2206 2HCl inhibited MMP-9 expression in 4NQO-induced tongue tumors. (b) MK2206 2HCl inhibited RhoC expression in 4NQO-induced tongue tumors. All data are presented as the mean ± SD. ^∗∗^*P* < 0.01 as compared with the blank group, ^##^*P* < 0.01 as compared with the 4NQO+captisol group. Magnification ×400. (c) MK2206 2HCl inhibited the RNA expression of MMP-9 and RhoC in CAL27 cells. (d) MK2206 2HCl inhibited the RNA expression of MMP-9 and RhoC in SCC25 cells. All data are presented as the mean ± SD. ^∗∗^*P* < 0.01,  ^∗^*P* < 0.05 as compared with the control group. (e) MK2206 2HCl inhibited the protein expression of MMP-9 and RhoC in OSCC cells.

To further detect the mechanism of the chemopreventive effect of MK2206 2HCl, we also detected MMP-9 and RhoC (key regulators of tumorigenesis) expression in OSCC cells and 4NQO-induced mouse tongue cancers through RT-PCR, western blotting, and IHC. *In vitro*, CAL27 and SCC25 cells treated with MK2206 2HCl for 24 h downregulated the RNA and protein expression of MMP-9 and RhoC, as shown by real-time PCR (Figures 12(c) and 12(d)) and western blotting (Figure 12(e)). In addition, compared with the 4NQO+captisol group, MK2206 2HCl also significantly reduced MMP-9 and RhoC expression in 4NQO-induced mouse tongue cancers in the 4NQO+MK2206 2HCL group via IHC staining (Figures 12(a) and 12(b)).

These results indicated that the chemopreventive effect of MK2206 2HCl may be through regulating MMP-9 and RhoC expression in OSCC.

## 4. Discussion

OSCC is an advanced form of precancerous lesions, and early chemoprevention may provide the best strategy to inhibit cancer progression. It has been reported that most cancers have hyperactivation of AKT, making it an attractive molecular target. Through TCGA database analysis, we found that AKT was highly expressed in OC. Studies have shown that MK2206 2HCl is one of the few AKT inhibitors used in the treatment of head and neck tumors [23], but its dose toxicity limits its application. In this study, we first detected the potential chemopreventive effect of MK2206 2HCl on a 4NQO-induced mouse tongue carcinogenesis model. Our findings demonstrated that MK2206 2HCl could markedly reduce the incidence rate and growth of OC and inhibit the transformation of dysplasia to cancer in the 4NQO-induced mouse tongue carcinogenesis model, and all mice were tolerated during the entire treatment well. In addition, our results also showed that MK2206 2HCl could inhibit the proliferation, colony formation, invasion, and migration abilities of OSCC cells *in vitro*.

MK2206 2HCl inhibited the dysplasia of tongue mucosal epithelium in the 4NQO-induced mouse tongue carcinogenesis model. p21 is a cell cycle arresting protein that correlates with the ability of cells to proliferate. p-Rb is a cell cycle checkpoint. And Ki67 is called the proliferation index, which represents the active degree of cell proliferation. Our IHC results showed that MK2206 2HCl inhibited the expressions of p-AKT and Ki67 and promoted the expression of p21 in mouse tongue cancers. In addition, MK2206 2HCl induced cell cycle arrest in OSCC cells *in vitro*, and further detection showed that MK2206 2HCl inhibited the expression of p-Rb and promoted the expression of p21. Specifically, MK2206 2HCl inhibited OSCC cell proliferation and was associated with G2/M cell cycle accumulation. It is reported that apoptosis may kill misplaced cells. Thus, apoptosis serves as an important process for inhibiting metastasis and proliferation [24]. Our results found that MK2206 2HCl promoted apoptosis in OSCC cells. Studies have shown that MK2206 2HCl could promote MCL-1 and cleaved-caspase 3 by inhibiting AKT phosphorylation [25]. MCL-1 and cleaved-caspase 3 play an important role in the process of apoptosis [26]. Thus, cell proliferation is closely related to cell cycle and apoptosis.

In our study, MK2206 2HCl promoted LC3 II expression. High expression of LC3 is considered to be one of the most reliable markers of autophagy [27]. Remarkably, autophagy, a vital process, could promote the survival of normal cells and trigger the death of harmful cells [28]. Promoting the occurrence of autophagy could inhibit the proliferation of cancer cells in colorectal cancer, which was consistent with our results [29]. Studies have shown that the anticancer activity of anticancer drugs is attributable to triggering autophagy and apoptosis [30]. Interestingly, the PI3K/AKT signaling pathway plays a pivotal role in regulating the interaction between apoptosis and autophagy [31]. Targeting the PI3K/Akt/GSK3 pathway could promote autophagy and impose sensitivity of cancer cells to apoptosis [32]. Inhibition of p-AKT expression could induce autophagy and apoptosis of human OC cells [33]. The complex crosstalk between apoptosis and autophagy is mediated by ATG5, BCL-2, and Beclin-130-32 [34, 35]. In this study, we also found that MK2206 2HCl could decrease p-AKT expression and promote autophagy and apoptosis. Therefore, we speculate that the chemopreventive effect of MK2206 2HCl on the 4NQO-induced mouse tongue carcinogenesis model may be related to promoting autophagy and apoptosis.

In addition, we found that MK2206 2HCl could also inhibit angiogenesis and MC infiltration in 4NQO-induced mouse tongue cancers. Uncontrolled cell growth and metastasis are important features of malignant tumors, and these processes are closely related to angiogenesis. Studies have found that the knockdown of AKT1 in mice could affect endothelial cell migration and NO release, resulting in a decrease in VEGF-mediated angiogenesis [36]. MMP-9 is also related to angiogenesis by regulating the deposition of tumor angiogenesis factors and proangiogenic factors, resulting in the recruitment of vascular endothelial cells [37]. Furthermore, MC are innate immune cells inherent in tissues, which can promote angiogenesis and accelerate the progression of malignant tumors by releasing different chemokines (such as TNF-*α* and IL-1b) and inflammatory cytokines [38].

Although many reports on the tumor suppressor mechanism of MK2206 2HCl have been published, it is still unclear how MK2206 2HCl regulates the progression of OC in the mouse 4NQO chemoprevention model. In addition to the occurrence of autophagy, we also found that the tumorigenesis-related factor (MMP-9 and RhoC genes) expression was decreased *in vitro* and in 4NQO-induced mouse tongue cancers in this study. First, MMP-9 could promote tumorigenesis by degrading the extracellular matrix and basement membrane. Its promoter region contains the activated protein AP-1 [39]. Studies have indicated that PI3K/AKT signaling axis activation could activate AP-1 and induce the expression of MMP-9, ultimately promoting tumor cell proliferation, invasion, and migration [40–42]. In addition, RhoC is also a key mediator in the proliferation and metastasis of tumor cells, and AKT could regulate RhoC expression [43]. The lack of RhoC could inhibit the metastasis of tumor cells by regulating the interaction between tumor cells and endothelial cells [44]. Interestingly, in our previous study, MMP-9 promoted OSCC cell proliferation and metastasis by regulating RhoC expression [45].

Various studies have found that autophagy plays a direct role in tumor cell invasion and movement by regulating extracellular matrix proteins [46]. Autophagy regulates focal adhesion dynamics during cell migration and invasion [47]. Focal adhesion kinase (FAK) leads to the activation of SRC kinases and paxillin during cell detachment from the substrate and inhibits autophagy, thereby promoting cell metastasis [48, 49]. Our previous study also found that MMP-9 positively regulates the expression of RhoC, and Rho and Rac1 regulate cell motility by promoting the formation of cell membrane protrusions, lamellipodia, and filopodia. There is also a relationship between the regulation of cell migration and autophagy by the Rho family. The ability of Rho to induce blood cell migration in Drosophila depends on autophagy-related gene 1 and p62 [50].

Based on the above, we preliminarily speculate that the chemopreventive effect of MK2206 2HCl on the 4NQO-induced mouse tongue carcinogenesis model may also be related to the MMP-9/RhoC signaling pathway and autophagy.

## 5. Conclusion

In summary, our findings first demonstrated the chemopreventive effect of the AKT inhibitor MK2206 2HCl on the 4NQO-induced mouse tongue carcinogenesis model, which may be mediated by inhibiting tumor growth, angiogenesis, and MC infiltration and through suppressing the MMP-9/RhoC signaling pathway, autophagy, and apoptosis. These findings preliminarily provide basic support for the development of MK2206 2HCl as a chemopreventive drug for OC.

## Figures and Tables

**Table 1:** Chemopreventive effect of MK2206 2HCl on 4NQO-induced mouse oral carcinogenesis.

	Mouse no.	Visible tumor	No. of tumor	Tumor volume (mm^3^)	Normal mucosa	Mild to moderate dysplasia	Severe dysplasia	Cancer
Blank	20	—	—	—	20 (100%)	—	—	—
4NQO+captisol	18	17 (94.4%)	35	4.05 ± 5.83	—	3 (16.7%)	2 (11.1%)	13 (72.2%)
4NQO+MK2206 2HCl	19	11 (57.9%)^∗^	17	3.01 ± 2.88	—	4 (21.1%)	8 (42.1%)	7 (36.8%)^∗^

^∗^ *P* < 0.05 as compared with the 4NQO+captisol group.

## Data Availability

All data generated or analyzed during this study are included in this published article.

## Abbreviations

OC: Oral cancer
OSCC: Oral squamous cell carcinoma
4NQO: 4-Nitroquinoline-1-oxide
MC: Mast cell
IHC: Immunohistochemistry.

## Supplementary Materials

Supplementary Materials Supplemental Figure 1: MK2206 2HCl induced LC3 II and p62 expressions in OSCC cells. (a) MK2206 2HCl promoted LC3 II expression in SCC25 cells as showed by immunofluorescence. Magnification ×400. (b) MK2206 2HCl decreased p62 expression in SCC25 cells. All data are presented as the mean ± SD. ^∗∗^*P* < 0.01 as compared with the control group. Magnification ×400. (c) The level of LC3 II was increased by western blotting in MK2206 2HCl-treated SCC25 cells.
