# Supplementary material for: Chemoprevention of 4NQO-Induced Mouse Tongue Carcinogenesis by AKT Inhibitor through the MMP-9/RhoC Signaling Pathway and Autophagy
Source: Anal Cell Pathol (Amst). 2022 Oct 5;2022:3770715. doi: 10.1155/2022/3770715 (PMC9556259; doi:10.1155/2022/3770715)
Supplement: Supplementary Materials — Supplemental Figure 1: MK2206 2HCl induced LC3 II and p62 expressions in OSCC cells. (a) MK2206 2HCl promoted LC3 II expression in SCC25 cells as showed by immunofluorescence. Magnification ×400. (b) MK2206 2HCl decreased p62 expression in SCC25 cells. All data are presented as the mean ± SD. ∗∗P < 0.01 as compared with the control group. Magnification ×400. (c) The level of LC3 II was increased by western blotting in MK2206 2HCl-treated SCC25 cells. [file 3770715.f1.docx]

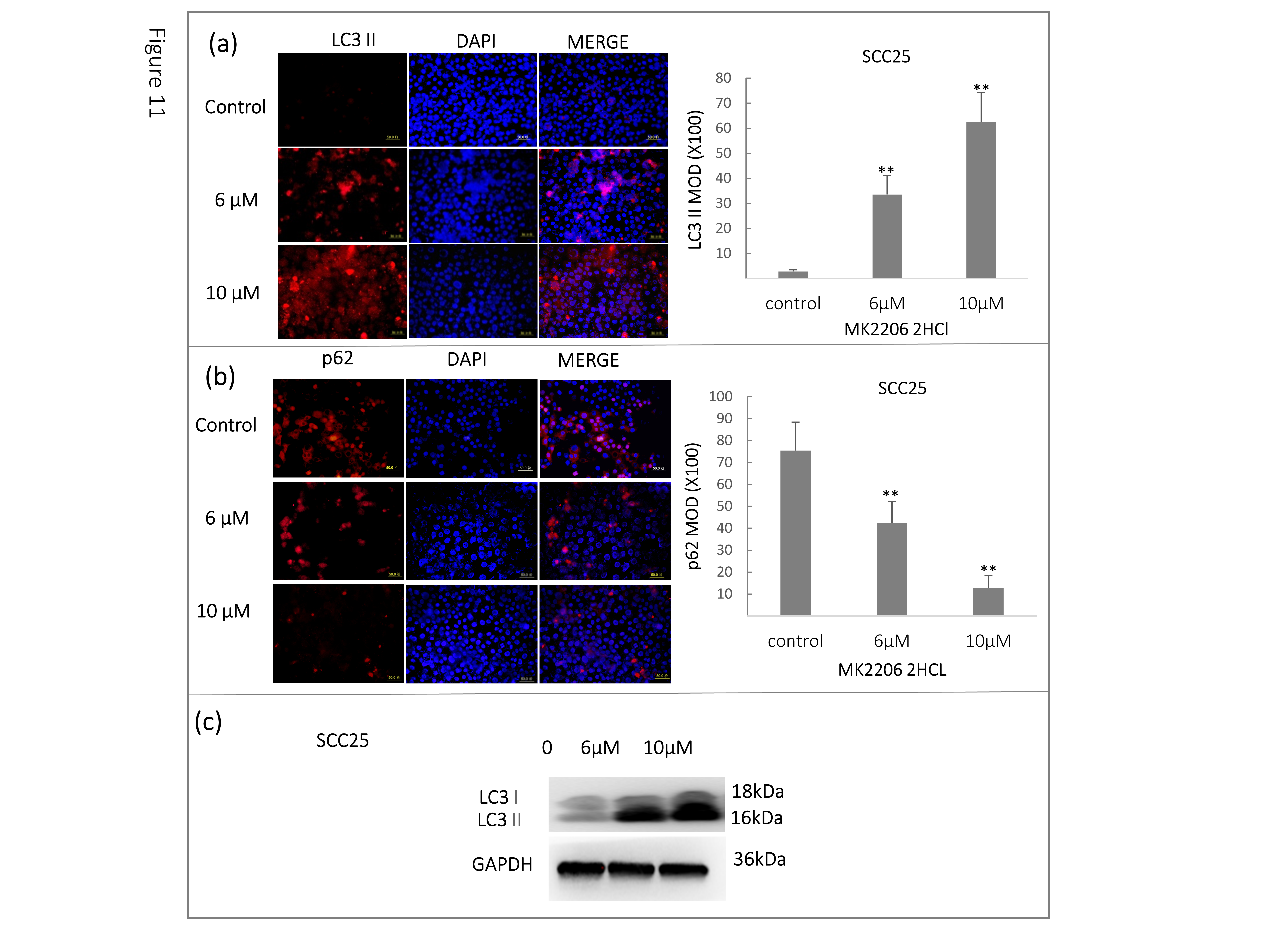


Supplemental figure 1. MK2206 2HCl induced LC3 II and p62 expressions in OSCC cells.

(a) MK2206 2HCl promoted LC3 II expression in SCC25 cells as showed by immunofluorescence. Magnification x400. (b) MK2206 2HCl decreased p62 expression in SCC25 cells. All data are presented as the mean ± SD. ^**^*P*<0.01 as compared with the control group. Magnification x400. (c) The level of LC3 II was increased by western blotting in MK2206 2HCl treated SCC25 cells.
